# Instruments to assess the perception of physicians in the decision-making process of specific clinical encounters: a systematic review

**DOI:** 10.1186/1472-6947-7-30

**Published:** 2007-10-15

**Authors:** France Légaré, David Moher, Glyn Elwyn, Annie LeBlanc, Karine Gravel

**Affiliations:** 1 Research Centre of the Centre Hospitalier Universitaire de Québec, Québec, Canada; 2 Department of Family Medicine, Université Laval, Québec, Canada; 3 Department of Epidemiology & Community Medicine, Faculty of Medicine, University of Ottawa, Ottawa, Canada; 4 Department of General Practice, Centre for Health Sciences Research, Cardiff University, Cardiff, Wales, UK

## Abstract

**Background:**

The measurement of processes and outcomes that reflect the complexity of the decision-making process within specific clinical encounters is an important area of research to pursue. A systematic review was conducted to identify instruments that assess the perception physicians have of the decision-making process within specific clinical encounters.

**Methods:**

For every year available up until April 2007, PubMed, PsycINFO, Current Contents, Dissertation Abstracts and Sociological Abstracts were searched for original studies in English or French. Reference lists from retrieved studies were also consulted. Studies were included if they reported a self-administered instrument evaluating physicians' perceptions of the decision-making process within specific clinical encounters, contained sufficient description to permit critical appraisal and presented quantitative results based on administering the instrument. Two individuals independently assessed the eligibility of the instruments and abstracted information on their conceptual underpinnings, main evaluation domain, development, format, reliability, validity and responsiveness. They also assessed the quality of the studies that reported on the development of the instruments with a modified version of STARD.

**Results:**

Out of 3431 records identified and screened for evaluation, 26 potentially relevant instruments were assessed; 11 met the inclusion criteria. Five instruments were published before 1995. Among those published after 1995, five offered a corresponding patient version. Overall, the main evaluation domains were: satisfaction with the clinical encounter (n = 2), mutual understanding between health professional and patient (n = 2), mental workload (n = 1), frustration with the clinical encounter (n = 1), nurse-physician collaboration (n = 1), perceptions of communication competence (n = 2), degree of comfort with a decision (n = 1) and information on medication (n = 1). For most instruments (n = 10), some reliability and validity criteria were reported in French or English. Overall, the mean number of items on the modified version of STARD was 12.4 (range: 2 to 18).

**Conclusion:**

This systematic review provides a critical appraisal and repository of instruments that assess the perception physicians have of the decision-making process within specific clinical encounters. More research is needed to pursue the validation of the existing instruments and the development of patient versions. This will help researchers capture the complexity of the decision-making process within specific clinical encounters.

## Background

Practising medicine involves making decisions at all stages of the clinical process [1]. Although a great deal of varied terminology is used to describe doctors' thinking, the term "decision-making process" is used extensively in the medical and healthcare literature [2]. The decision-making process is broadly defined as global judgements by a clinician about the appropriate course of action and is said to be unspecified, as a number of processes may produce a decision [3]. In clinical settings, it is also understood as the use of diverse strategies to generate and test potential solutions to problems that are presented by patients and involves using, acquiring and interpreting the indicators and then generating and evaluating hypotheses [4]. Processes or strategies that will be used may be based on what the clinician was taught, his or her own representation of the evidence supporting each course of action, or the prevailing practice in a given institution [4].

In recent years, there has been a growing interest in new representations of the clinical decision-making process that better address its complexity within specific clinical encounters. Indeed, providing medical care to a patient is now increasingly considered a dynamic and interactive process known as "shared decision-making" [5-7]. Characteristics of shared decision-making include that at least two participants, clinician and patient, be involved; that there be a two-way exchange not only of information but also of treatment preferences; that both parties take steps to build a consensus about the preferred treatment; and that an agreement be reached on the treatment to be implemented [5]. Shared decision-making includes the following components: establishing a context in which patients' views about treatment options are valued and deemed necessary, transferring technical information, making sure patients understand this information, helping patients base their preference on the best evidence; eliciting patients' preferences, sharing treatment recommendations, and making explicit the component of uncertainty in the clinical decision-making process [8].

Shared decision-making does not exclude a consideration of the values and preferences of the physician and occurs through a partnership in which the responsibilities and rights of each of the parties and the benefits for each party are made clear [9]. Given the recognition that patient-physician interactions and by extension, clinical decision-making processes, are dynamic and reciprocal in their nature, it is surprising to find little systematic evaluation of the physicians' perspective of this entity [10]. Consequently, there has been a renewed interest in capturing the perspective of physicians of the decision-making process within specific clinical encounters. Therefore, the aim of this systematic review was to identify instruments that assess the perception of physicians of the decision-making process within specific clinical encounters.

## Methods

### Search strategy

Covering all years available (to April 2007), we conducted an electronic literature search of the following databases: PubMed, PsycINFO, Current Contents, Dissertation Abstracts and Sociological Abstracts. Three information specialists were consulted to help develop, update and run the search strategy. The following MeSH terms and free text words were used to create specific search strategies for each database: "decision making", "physicians", "health personnel", "doctors", "practitioners", "health personnel attitudes", "measurement", "questionnaire", "psychometrics" and "psychological tests". We included titles of publications and their respective abstract in English or French that potentially included an eligible instrument. Initially, if a dissertation abstract was found along a publication, both were kept. We also contacted 10 experts in the field (list available from authors) and contacted corresponding authors of included instruments. Lastly, we reviewed bibliographies of the included instruments. Once we included an instrument, we conducted an electronic search of the first author.

### Selection criteria

All of the searches were downloaded to a reference database for initial screening of titles and abstracts by a single member of the review team. Prior to screening, duplicates were removed from the database. Titles of publications and their respective abstract reporting editorials, letters, surveys, clinical vignettes or the completion of an Objective Structured Clinical Examination or the evaluation of a simulated patient were excluded. After the initial screening, if detailed information about the titles of publications and their respective abstract was questionable, the full text of these publications was sought. Then, two reviewers independently appraised these publications to identify ones that reported on the use or development an eligible instrument. Discrepancies between the two reviewers were resolved through discussion.

### Identification of eligible instruments

The following inclusion criteria were applied: 1) a self-administered instrument was presented; 2) the instrument evaluated the perspective of physicians, including residents, of the decision-making process within specific clinical encounters, 3) the collection of data occurred after a specific clinical encounter in a 'real' clinical setting; 4) the report included sufficient description to permit critical appraisal of the instrument (for example, the instrument was provided as an appendix or we were able to get a copy from the author); and 5) there were quantitative results following the administration of the instrument. An instrument was defined as a systematic procedure for the assignment of numbers to aspects of objects, events or persons as indicated by its construction, administration and scoring procedure according to prescribed rules [11].

The outcomes of interest included the perception of physicians of the decision-making process within specific clinical encounters as well as the outcome of the decision itself such as satisfaction with the decision. The decision-making process was defined in an inclusive manner as global judgements by a physician about the appropriate course of action [3]. An instrument was deemed eligible if one of its sub-scales or some of its items tapped into the outcomes of interest.

### Data extraction

The data extraction form, derived from McDowell (1987) [12], covered characteristics of the source of information and characteristics of the instrument itself, such as name of the instrument, origin of first author, main purpose, description of the instrument, characteristics of the response scale, presence of a corresponding patient instrument, development procedures, conceptual/theoretical foundation, validity, reliability (e.g. internal consistency) and responsiveness of the instrument.

A conceptual framework was considered to be used if the author referred to a set of concepts and the propositions that integrate them into a meaningful configuration [13]. A theory was deemed to be used if the author referred to a theory, defined as a series of statements that purport to account for or characterize some phenomenon with a much greater specificity that a conceptual framework [13]. Otherwise, the nature of the source of references used by the author was used to identify a broad conceptual basis.

Content validity (i.e. the extent to which all relevant aspects of the domain or area that is being measured are represented in the instrument), construct validity (i.e. the extent to which the instrument relates to other tests or constructs in the way that was expected) and criterion validity (i.e. the extent to which the instrument relates to a gold standard to which it is compared) were also assessed [14]. Responsiveness (i.e. the extent to which the instrument measured change within persons over time) was also assessed [15].

Using the Science Citation Index, we assessed how many times the included instruments had been cited in subsequent published research in French or English. Lastly, for each instrument, we assigned one main evaluation domain defined as a subjective interpretation by the reviewers of the main construct that the instrument was assessing. Sources of disagreement were discussed and resolved by consensus and only consensus data was used. Data extraction was completed by two members of the team.

### Quality assessment

The quality of reporting of the included studies was assessed by two reviewers independently, using a modified version of the following instrument, Standards for Reporting of Diagnostic Accuracy (STARD) [16-18]. The original STARD contains 25 items pertaining to study question, study participants, study design, test methods, reference standard, statistical methods, reporting of results and conclusions. However, because we were interested in instruments assessing the perception of physicians of the decision-making process within specific clinical encounters, we added one more item under the section "statistical methods." This new item assessed if the authors of the included instrument had taken into account that one physician could only contribute to one questionnaire for the statistical analyses used to provide evidence on its reliability and validity (i.e., the non-independence of data). For each instrument, we chose one main study. In instruments for which more than one report was included, we chose the one that reported the most details on the development and psychometrics of the instrument in its most recent version.

## Results

### Included instruments

**Figure 1:**
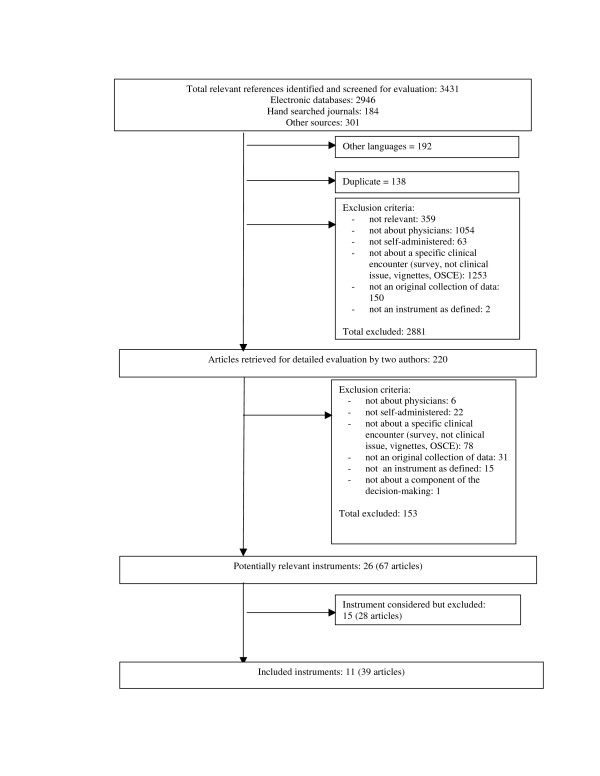
Progress through the stages of the systematic review.

The initial search resulted in 3431 records (Figure 1). From these, 192 records that were in a language other than French or English, and 138 duplicates were removed. After applying our eligibility criteria, 218 full text articles were retrieved for detailed evaluation. Twenty-six instruments (67 articles) were potentially eligible of which a further 15 (28 articles) were excluded because they were not designed to collect data for a specific clinical encounter [19-46]. Therefore, 11 instruments (39 articles) were included [47-85]. We were able to get access to a published version or a copy of all included instruments.

### Characteristics of the included instruments

Overall, the included instruments were published between 1986 and 2007 (Table 1) [47-85]. Nine instruments were developed in North America and available in English [47-67,69-72,74-85]. Among these, three were available in French [57,58,64-67,69-71,77-82]. Two instruments were developed in Europe [68,73]. For most instruments, the first author was affiliated with a Faculty of Medicine or a medical organisation (n = 8) [54-59,64-72,74,76-85], followed by a School of Nursing (n = 1) [47-53,75], Department of Communication (n = 1) [60-63] and Research Group in Psychosomatic Rehabilitation (n = 1) [73]. Most instruments were developed for non-specific clinical problems (n = 9) [54-74,76,83-85]. One instrument was developed for intensive care unit-related problems [47-53,75] and one for inflammatory bowel diseases [77-82].

**Table 1:** Characteristics of the 11 included instruments

**Name of the instrument (First author, year of publication)**	**Origin of first author**	**Main purpose (Measurement aim, clinical domain and context of use envisioned by author)**	**Description (number of dimensions and items)**	**Response scale**	**Patient version**	**Number of citations**
Physician Satisfaction Scale (Shore, 1986) [54, 76, 83]	Department of Preventive, Family and Rehabilitation Medicine	-To study physician satisfaction in encounter-specific situations. -Non-specific clinical problem. -Clinical and educational (the authors thought that the use of this instrument could serve as a possible pathway to changing providers' behaviour through self-awareness).	-2 dimensions/16 items -understanding the patient's problem, having a sense that the patient understood what the physician said, affective reactions to the interaction with the patient and satisfaction of physician and patient were included.	5 pt Likert	No	14
Mental Work-Load Instrument (Bertram, 1992) [55, 56, 59, 74]	Department of Social and Preventive Medicine	-To assess the subjective experience or cost incurred by a physician in performing patient care tasks that reflect the combined effect of demands imposed by task requirements, the support personnel, information and equipment resources provided the physician's skill and experience, strategies adopted by the physician, effort exerted, and emotional responses to the situation. -Non-specific clinical problem. -Quality improvement (the authors aimed at taking into account the cognitive processes involved in physician work so that physicians could be trained or patient care settings structured to minimize the physician limitations and improve their performance as well as the productivity of the organization).	-5 dimensions/6 items -mental effort, physical effort, difficulty, performance and psychological stress (each with 1 item except performance with 2).	0.0 – 10.0 visual analogue scale with bipolar descriptors.	No	8
Questionnaire concerning the sources of frustration physicians experience in their work with patients (Levinson, 1993) [72]	Department of Medicine	-To identify specific aspects of patient visits that cause physician frustration and to develop a self-assessment instrument for physicians -Non-specific clinical problem. -Quality improvement (the authors thought that through reflection, this instrument would assist physicians to identify areas of their experience with patients that are frustrating and that need improvement and that ultimately, patient care would be improved).	-7 dimensions/25 items -lack of trust, too many problems, feeling distressed, lack of adherence, lack of understanding, demanding/controlling patients, and special problems (each with 3–4 items).	5 pt Likert	No	49
Physician Satisfaction Questionnaire (Suchman, 1993) [84, 85]	Department of Medicine and Psychiatry	-To assess physician satisfaction with primary care office visits in encounter-specific contexts, and to identify determinants of physician satisfaction. -Non-specific clinical problem. -Research, clinical and educational (the authors thought that this instrument could be used to guide the preparation of future physicians with skills, knowledge and attitudes they will need to practice in a manner that is satisfying both to their patients and to themselves).	-4 dimensions/20 items -quality of the patient doctor relationship, adequacy of the data collection process during the visit, appropriate use of time during the visit and patient's non-demanding, cooperative nature.	5 pt Likert	No	44
Collaboration and Satisfaction about Care Decisions (Baggs, 1994) [49–53, 75]	School of Nursing	-To measure nurse-physician collaboration in making specific patient care decisions in intensive care units. -Intensive Care Unit (ICU) settings (the author assumed that it could be used in non-ICU settings or to refer to other type of patient care decisions as well). -Research and quality improvement (the author thought ultimately, responses to this instrument could be linked to patient and provider outcomes).	-2 dimensions/9 items -level of collaboration between the physician and the nurse in making the decision (7 items) and satisfaction with the decision and decision-making process (2 items)	7 pt Likert	No	20
Medical Communication Competence Scale (Cegala, 1998) [60–63]	Department of Communication	-To measure doctor's and patient's perceptions of self and other communication competence during a general medical interview. -Non-specific clinical problem. -Research.	-4 dimensions/37 items -information giving, seeking and verifying and socio emotional communication.	7 pt Likert	Yes	10
Provider Decision Process Assessment Instrument (Dolan, 1999) [57, 64–67, 69–71]	Department of Medicine	-To measure physicians' degree of comfort with a clinical treatment decision. -Non-specific clinical problem. -Quality improvement and research (The author asserts that combining it with an equivalent patient-oriented measure would make it possible to comprehensively assess the clinical decision making process).	-4 dimensions/12 items -uncertainty, knowledge, value, effectiveness. Note: the English version of the questionnaire was translated into French by a professional translator and then back-translated into English by a family physician who was not associated with the authors.	5 pt Likert	Yes	7
Patient-Physician Discordance Scale (Sewitch, 2003) [77–82].	Department of Medicine	-To assess discordance between physicians and their patients on evaluations of health-related information. -Chronic diseases, most specifically inflammatory bowel diseases. -Clinical and research.	-3 dimensions/10 items -symptoms and treatment, well-being and communication and satisfaction. Note: the English version of the questionnaire was translated into French by an independent bilingual medical translator and a bilingual psychology student, and then back-translated into English by two other bilingual graduate students who were not associated with the authors.	100-mm visual analogue scale	Yes	9
Mutual Understanding Scale (Harmsen, 2005) [68]	Department of Health policy and management and Department of general practice	-To develop a reliable measure of mutual understanding between general practitioners and patients. -Non-specific clinical problem. -Research or professional training.	-3 dimensions/8 criteria -perception of one's own ability to explain to the patient, perception of the patient's ability to explain to the physician, and perception of patient's understanding of consultation aspects.	Mixed	Yes	1
Reasons for Treatment Selection Questionnaire (Linden, 2006) [73]	Research Group Psychosomatic Rehabilitation	-To assess reasons why physicians select or do not select a certain treatment. -Non-specific clinical problem. -N/A	-5 dimensions/22 items -theoretical knowledge, experiential knowledge, situational knowledge, anticipations about the further course of treatment, and interactional knowledge	5 pt categorical response scale	N/A	0
Questionnaire concerning the doctor-patient communication skills (Campbell, 2007) [58]	Royal College of Physicians and Surgeons of Canada	- To develop and psychometrically assess the feasibility, reliability and validity of an assessment tool in which both doctor and patient perceptions of the communication that occurred in a single office visit are captured. - Non-specific clinical problem in general practice and medical specialists practice. - Designed for use in the office settings.	- 2 dimensions/19 items - The final instrument captures both the process aspects of the visit (e.g. patient greeting, listening, and understanding) as well as the content of the visit (e.g. explanations, treatment options, next steps).	5 pt Likert	Yes	0

N/A: Information is not available in publications in French or English

All instruments were multi-dimensional. The mean number of items per instrument was 16.7 (range: 6 to 37). Seven instruments used a Likert response scale [47-54,57,58,60-67,69-72,75,76,83-85], two used a Visual Analog Scale [55,56,59,74,77-82] and one used a 5-point categorical response scale [73]. One instrument used a mix of response scales [68]. Five instruments offered a patient version [57,58,60-71,77-82].

Based on the Science Citation Index, nine instruments had been cited at least once in subsequent research published in French or English with the older ones being more likely to be cited more often (Spearman r = -0.68; p = 0.03).

### Development procedures and psychometrics of the included instruments

Authors of nine instruments reported on their explicit use of some conceptual framework or broad conceptual domain (Table 2) [47-53,55-75,77-82]. For seven instruments, we were able to find evidence of their validity and reliability [47-67,69-71,74-76,83-85]. For two instruments, evidence of validity and reliability data was available only for the combined use of the physician's and patient's questionnaires [68,77-82]. For one instrument, we could not find evidence of validity and reliability data published in French or English [73].

**Table 2:** Development and psychometric properties of the 11 included instruments

**Instrument**	**Origins and development**	**Conceptual framework**	**Validity**	**Reliability**
Physician Satisfaction Scale (Shore, 1986) [54, 76, 83]	Delphi method with family physicians to develop first 43-item version on 4 sub-scales. Tested on 49 physicians. The scale was then reduced to 16 items on two sub-scales and tested back on 131 physicians from Family Medicine, General Internal Medicine and Paediatric programs.	Not clear	Content validity: -efforts were put in the development phase of the instrument to ensure validity of the items (consultation with Delphi method). Construct validity: -factor analysis confirms two factors (average loading for patient-related: 0.71 and average loading for contextual: 0.58) -the instrument did not discriminate between different residency programs, geographical location or years of training.	Internal consistency: -Cronbach alpha for global scale: 0.85 (patient-related subscale: 0.89 and contextual subscale: 0.63)
Physician Mental Workload (Bertram, 1992) [55, 56, 59, 74]	A previous version of the instrument was constructed through discussion with physicians and from a preliminary literature search. It was tested in two different hospital settings and revisions led to a 10-item version also presented on a visual analogue scale. The present instrument is a 6-item adaptation of this previous one. It was tested on 22 residents, who in all saw a total of 92 patients during an afternoon clinic session. It was tested with residents and physicians in practice, internal medicine and very few in paediatric residency	Broad domain of human performance research and measurement approaches employed in the field of human factors research. It encompasses motivational, social, attitudinal, and organizational factors as well as human capability assessment, information processing and decision making and stress effects on performance.	Content validity: -efforts have been made in the development of the first version of the instrument to ensure face validity by consulting physicians, and formal content validity by literature review. The process of selection of the items included in the present version is not described. Construct validity: -correlates with: fatigue: r = 0.42, mean experience, r = -0.65, resident self-rated quality: r = -0.67, third observer's overall quality rating, r = -0.18, personal interaction factor score: r = -.04, technical performance factor score: r = -0.38 -does not correlate with: total number of patients seen, proportion of new patients, patient complexity, personal interaction performance, overall ratings by faculty members and age of the residents. -does not discriminate between female and male residents nor among postgraduate years. Note: In order to not violate the assumption of independence between observations, the unit of analysis chosen was the resident, and an associated average score per resident was used with patient-specific measures.	Internal consistency : -Cronbach alpha: 0.80 (unadjusted for non-independence of observation) -Inter-items correlation: mean: 0.45 (SD.: 0.19)
Physician Frustration in Communicating with patients (Levinson, 1993) [72]	A group of experts developed an initial set of 32 items corresponding to common problems encountered by physicians in their encounters with patients. This was pilot-tested on 107 physicians of diverse trainings. A second version of 42 items on 8 sub-scales was distributed to 931 physicians, and was reduced to 39 items, and this version was completed by 1076 physicians. Final version consists of 25 items on 7 sub-scales.	Broad domain pertaining to the quality of the communication and the relationship between patients and their physicians as important pathways to both the medical outcome and satisfaction of both parties.	Content validity: -efforts were put into the development phase of the instrument to ensure validity of the items (x consultation of experts) Construct validity: -factor analysis confirms 7 factors. Mean respective factor loading for all items is 0.68 (SD = 0.10) -the instrument discriminated between younger and older physicians (i.e. younger physicians had higher scores on all subscales meaning they felt more frustrated than the older physicians) and between primary care physicians and specialists on two subscales: too many problems and feeling distressed (i.e. primary care physicians had higher score than specialists). Greater time spent in primary care was associated with higher scores on several subscales. Convergent validity was shown with physicians' general level of satisfaction and the percentage of visits they reported as being frustrating correlating with higher scores on most subscales.	Not provided
Physician Satisfaction with Primary Care Office Visits (Suchman, 1993) [84, 85]	The development of this instrument was achieved within a larger initiative, "The Collaborative Study of Communication Dynamics". This initiative was organized by the Task Force on Doctor and Patient of the Society of General Internal Medicine that was conducted at 11 sites in North America. Members of this group included well-known experts in the field of patient-doctor interaction and communication. The instrument was tested with 124 physicians (35 residents, 60 general internists and 3 family physicians) who saw a total of 550 patients.	Not clear	Content validity: face validity is considered in that the items of the scale share common ground with previously published measures Construct validity: -factor analysis reveals 4 distinct factors, but since these construct domains were not predicted at first in a theoretical framework, this analysis provides weaker support for the construct validity of the instrument -a number of patient characteristics were significantly associated with the sub-scales. For example, emotional distress of patients was negatively correlated with all satisfaction dimensions except the time dimension. Satisfaction with the patient doctor relationship sub scale was the most important determinant of global satisfaction (R^2 ^= 39%) while the adequacy of data collection process was the second most important determinant (R^2 ^= 4%). Note: Non-independence of observations was taken into account: a bootstrapping technique was used to create 10 replication samples of n = 124 and factor analysis was then performed 10 times.	Internal consistency -Cronbach alpha for the 19 specific items (excluding the general satisfaction question): 0.82 -Cronbach alpha for all 20 items: 0.84
Collaboration and Satisfaction about Care Decisions (Baggs, 1994) [49–53, 75]	This instrument is based on a conceptual model for collaboration for conflict resolution. It was developed from an initial 2-item version, the Decision About Transfer, a literature review on the subject and opinion of experts in collaborative practice and of practising professionals in the field. It was pilot tested on a convenience sample of 32 nurses and 26 residents in an intensive care unit.	Thomas (1976) conceptual model of collaboration for conflict resolution and organisational theory by Thompson (1967).	Content validity: -literature review on the subject and opinion of experts in collaborative practice and of practising professionals in the field. Construct validity: -factor analysis confirms a single factor (Eigen value of 4.5, no other higher than 1) that explains 75% of the 6 specific collaboration items variance. Mean factor loading for the six specific collaboration items was 0.87 (SD.: 0.04). -convergence of a combined score of the six specific collaboration items with a combined score of the two satisfaction items: r = 0.66 Criterion validity: -correlation of the six specific collaboration items with the global collaboration question: r = 0.87 Note: Non-independence of observations was taken into account: factor analysis was performed with a sample size of 56 (i.e. all independent data entry points) and confirmed one factor for collaboration	Internal consistency: -Cronbach's alpha: 0.93 -Inter-item correlations: 0.52 – 0.83
Medical Communication Competence Scale (Cegala, 1998) [60–63]	Post-interview questionnaires in clinical setting as well as self and other evaluation of communication competence by 15 family practice residents inspired the development of a first version of 56 items. Six physicians scored each item for their importance to communication competence during a medical consultation. Best items constituted the 37 items final version. A corresponding patient instrument was also pilot-tested concomitantly. Hence, these two instruments were pilot-tested with 65 doctors and 52 patients who provided a total of 117 data entries.	Extensive theoretical review supports the development of the scale.	Content validity: efforts were put into the development phase of the instrument to ensure validity of the items (face validity by consultation of potential users) Construct validity: -Factor analysis supports construct validity, but complete loading data is missing. -A cluster analysis using Euclidean distances among standardized item response was performed for each file. As hypothesized by the author, the results of both cluster analysis covered 4 clusters: information giving, information seeking, information verifying and socio emotional communication. -a series of research questions in which within-sample comparisons (i.e. comparisons made within the physician data file and the patient data file) and between-sample comparisons (i.e. comparisons made between the physician and the patient data files) were shown to be consistent with the literature on doctor-patient communication. For example, doctors rated their socioemotional competence higher than their competence in information exchange than in any of the other information subscales paralleling poor competence in information exchange observed in previous researches.	Internal consistency for the doctor's scale (Cronbach alpha's) - information giving: 0.86 - information seeking: 0.75 - information verifying: 0.78 - socio emotional communication: 0.90
Provider Decision Process Assessment Instrument (Dolan, 1999) [57, 64–67, 69–71]	Based on the construct of decisional conflict, this instrument is an adaptation of O'Connor's 16-item Patient Decisional Conflict Scale. Data were obtained on two sites from 14 residents, 7 physicians and one fellow in General Internal Medicine.	Ottawa Decision Support Framework.	Content validity: face validity assessed by asking participants for direct feedback. Construct validity: moderately confirmed by negative correlation with two satisfaction items: satisfaction with the decision (Spearman's r = -0.58) and assessment of the quality of the decision (Spearman's r = -0.52).	Internal consistency: -Cronbach alpha: 0.878 Note: In order to not violate the assumption of independence between observations, a bootstrapping approach was used. (i.e. 30 random samples consisting of one patient from each of seven physicians) Cronbach alpha was 0.90, 95%CI= 0.87 – 0.92.
Patient-Physician Discordance Scale (Sewitch, 2003) [77–82].	On the basis of a literature review, two domains were identified: patient's health status and the office visit. Two experts, a clinical psychologist and a gastroenterologist, were provided with a list of items recorded from the literature review and asked to select the top 10 items thought to be relevant to making treatment decision. A consensus was reached after a brief discussion.	Broad domain of patient-physician discordance.	Content validity: Based on the literature review and two experts. For construct and criterion validity, data are provided only for the combination of the physician's and patient's questionnaires	Data are provided only for the combination of the physician's and patient's questionnaires.
Mutual Understanding Scale (Harmsen, 2005) [68]	This instrument was developed based on Kleinman's theory, a method of phasing or structuring of consultations by the physician (S.O.A.P. method) and a consensus method of decision-making called the Nominal Group Technique or expert-panel meeting	Kleinman's theory about the influence of culturally determined views on health beliefs and the necessity for physician and patient to demonstrate these views by exchanging explanatory models during the consultation.	Content validity: By using questions about different consultation aspects, known as GP standard of structuring the consultation, the complete consultation was covered. For construct and criterion validity, data are provided only for the combination of the physician's and patient's questionnaires	Data are provided only for the combination of the physician's and patient's questionnaires.
Reasons for Treatment Selection Questionnaire (Linden, 2006) [73]	N/A	Action theory	N/A	N/A
Questionnaire concerning the doctor-patient communication skills [58]	This pair of instruments was developed based on the Patient Centered Care method [98] and theories in the field of communication. Its authors drew on existing instruments and the communication skills expertise of 2 members of the steering group to create the pair of instruments. The initial instruments were administered to 4 specialists and 3 family doctors in Ontario, Canada, who, along with their patients, provided feedback. The final pair of instruments was tested with 16 family doctors and 22 specialists from 3 Canadian provinces. These doctors recruited a total of 1881 patients.	Patient Centered Care method [98] and theories in the field of communication.	Content validity: based on existing instruments and the communication skills expertise of 2 members of the steering group to create the pair of instruments. Construct validity: -Factor analysis was performed by using the whole set of 38 items (19 items in the doctor's questionnaire plus 19 items in the patient's questionnaire) to ascertain whether the patient and doctor items were 2 separate factors. Then by examining the data for patient and doctor separately, the authors ascertained if the process and content items accounted for separate factors. The items on all 3 datasets (i.e. 19 items from the patient data alone, 19 items from the doctor data alone, and the combined dataset of 38 items) were separately intercorrelated using Pearson product) moment correlations.	Internal consistency: -Cronbach alpha for the doctor and patient questionnaires: 0.70 and 0.69, respectively. Number of patients per doctor required for a reliable assessment of the doctor's overall communication skills: - The *G *analysis provided a *G *= 0.98 and 0.40 (standard errors of 0.003 and 0.02) for doctors and patients, respectively.

N/A: Information is not available in publications in French or English

None of the instruments provided data on their responsiveness (i.e. the extent to which it measures change within a physician over time). Lastly, the main evaluation domain that was assigned to each instrument were: satisfaction with the clinical encounter (n = 2) [54,76,83-85], mutual understanding between the health professional and the patient (n = 2) [68,77-82], mental workload (n = 1) [55,56,59,74], frustration with the clinical encounter (n = 1) [72], nurse-physician collaboration (n = 1) [47-53,75], perceptions of communication competence (n = 2) [58,60-63], degree of comfort with a decision (n = 1) [57,64-67,69-71] and information on medication (n = 1) [73].

### Quality of the studies that reported on the included instruments

Overall, the mean number of items reported on the modified STARD was 12.4 (range: 2 to 18}(Table 3). During the development of four instruments, the authors used an analytical approach that took into account the non-independence of data [47-53,55-57,59,64-67,69-71,74,75,84,85]. For the Mental Work-Load Instrument, the authors used a mean score per physician to perform the correlation analyses [55,56,59,74]. For the Physician Satisfaction Questionnaire, the authors used a bootstrapping approach to perform the factor analysis [84,85]. For the Collaboration and Satisfaction about Care Decisions instrument, the authors restricted their sample size to one data entry per physician (n = 56) to perform most of their analyses [47-53,75] and for the Provider Decision Process Assessment Instrument, the authors used a bootstrapping approach to perform their reliability analyses (i.e. Cronbach alpha) [57,64-67,69-71].

**Table 3:** Quality assessment of the studies that reported on the included instruments based on the modified version of STARD * For this instrument, only one publication in English was found. This publication reported on the study of physicians that had used the instrument. Other publications pertaining to this instrument were in German

**Section and Topic**	**Item**	[83]	[56]	[72]	[84]	[50]	[61]	[65]	[81]	[68]	[73]*	[58]
**TITLE/ABSTRACT**	Identify the article as a study concerning a measuring instrument.	+	+	+	+	+	+	+	+	+	0	+
**INTRODUC-TON**	State the research questions or study aims, like developing or validating a measuring instrument.	+	+	+	+	+	+	+	+	+	0	+
**METHODS**												
*Participants*	Describe the study population: The inclusion and exclusion criteria, setting and locations where the data were collected.	+	+	+	+	+	+	+	+	+	0	+
	Describe the method of recruitment of the participants.	0	0	0	+	0	0	0	+	+	0	+
	Describe participant sampling: Was the study population a consecutive series of participants defined by the selection criteria in items 3 and 4? If not, specify how participants were further selected.	0	0	0	0	0	+	0	+	+	0	+
	Describe data collection: Was data collection planned before the use of the measuring instrument?	0	0	0	+	0	+	0	+	0	0	+
*Test methods*	Describe the reference standard criterion validity and its rationale.	0	0	0	0	0	0	0	+	0	+	0
	Describe technical specifications of material and methods involved including how and when measurements were taken, and/or cite references for measuring instrument.	+	+	+	+	+	+	+	+	+	0	+
	Describe definition of and rationale for the units, cut-offs and/or categories of the results of the instrument and the reference standard.	+	+	+	+	+	+	+	+	+	0	0
	Describe the number, training and expertise of the persons executing and reading the measuring instrument and the reference standard.	0	0	0	+	0	0	0	0	0	0	0
	Describe other tests or relevant information for the readers concerning the measuring instrument (subjective).	+	+	+	+	+	+	+	+	+	+	+
*Statistical methods*	Describe methods for calculating or comparing measures of reliability, validity, and the statistical methods used to quantify uncertainty (e.g. 95% confidence intervals)	+	+	0	+	+	0	+	+	+	0	+
	Describe methods for calculating test reproducibility, if done.	0	0	0	0	0	0	+	0	0	0	+
	Describe a method that takes into account non-independence of data (if applicable)	0	+	0	+	+	0	+	0	0	0	0
**RESULTS**												
*Participants*	Report when study was done, including beginning and ending dates of recruitment.	0	0	0	0	0	0	0	+	+	0	0
	Report demographic characteristics of the study population (e.g. age, sex, employment, recruitment centers).	+	+	+	+	+	+	0	+	+	0	+
	Report the number of participants satisfying the criteria for inclusion (a flow diagram is strongly recommended).	0	0	0	0	0	0	0	+	+	0	+
*Test results*	Report time interval from the measuring instrument to the reference standard, and any measures administered in between.	0	0	0	0	0	0	0	+	0	0	0
	Report distribution of severity of the situation being assessed (define criteria) in those with the target condition; other diagnoses in participants without the target condition	0	0	0	0	0	0	0	0	0	0	0
	Report a cross tabulation of the results of the measuring instrument (including indeterminate and missing results) by the results of the reference standard; for continuous results, the distribution of the test results by the results of the reference standard	+	0	+	+	+	+	+	+	+	0	0
	Report any adverse events from performing the measuring instrument or the reference standard	0	0	0	0	0	0	0	0	0	0	0
*Estimates*	Report estimates of accuracy and measures of statistical uncertainty (e.g. 95% confidence intervals).	+	+	+	+	0	+	+	+	+	0	0
	Report how indeterminate results, missing responses and outliers of the measuring instrument were handled.	0	0	0	0	0	+	+	0	0	0	0
	Report estimates of variability of accuracy between groups of participants, if done.	0	0	+	+	0	0	+	0	0	0	+
	Report estimates of test reproducibility, if done.	0	0	0	0	0	0	+	0	0	0	+
**DISCUSSION**	Discuss the clinical applicability of the study findings.	+	+	+	+	+	+	+	+	+	0	+

		11/26	11/26	11/26	16/26	11/26	13/26	15/26	18/26	15/26	2/26*	14/26

* For this instrument, only one publication in English was found. This publication reported on the study of physicians that had used the instrument. Other publications pertaining to this instrument were in German.

## Discussion

We believe that the results of this systematic review are important. First, they indicate that there is an interest expressed by clinicians, health services researchers and educators in assessing the perspective of physicians about the processes leading to a decision within specific clinical encounters. This is congruent with the increasing number of randomized trials and systematic reviews examining the efficacy of interventions designed to bring about a change in clinical practice [86]. However, most of these trials assessed a change in health professionals' behaviour without assessing the underlying decision-making process that lead to such behavioural change. This review provides a list of standardized measures of the physician perspective of the clinical decision-making process, an essential step prior to behavioural change. Moreover, most of the included instruments provided some account of their conceptual or theoretical underpinnings. This is important because more attention needs to be given to the combination of different theories that could help us understand professional behaviours [87-90]. Therefore, this review provides health services researchers and educators with a set of standardized and theory-driven instruments that have the potential to improve the quality of implementation studies and by extension our understanding of health professionals' behaviour changes.

Second, this review provides evidence that health services researchers are beginning to use a dyadic and relationship-centered approach to clinical decision-making [91-93]. In other words, health services researchers are moving from studying groups of patients and health professionals separately to studying both simultaneously. For example, five of the six most recently developed instruments had corresponding patient versions [57,58,60-71,77-79,81,82]. Moreover, for the authors of two of these instruments, evidence of validity and reliability data was available only for the combined use of the physician's and patient's questionnaires [68,77-82]. This observation suggests that, increasingly, the clinical decision-making process is perceived as not being dissociable from the complex aspects of interdependence occurring between the physician and the patient. Indeed, the patient-physician relationship is an important component of physicians' satisfaction with their job [93]. Physicians' judgements about their experience with individual patients both reflect and shape what takes place during office visits and beyond [84]. This symmetry supports empirically what has previously been described on the basis of personal needs, namely, that both the physician and the patient have the same human needs for connection which can be fulfilled in the clinical encounter [84]. Therefore, future research in the field of clinical decision-making should foster the use of patient and physician versions of a similar instrument. In line with the growing interest for shared decision-making, this may allow for a more comprehensive assessment of the complexity of the clinical decision-making process and thus of its dynamic and reciprocal nature [65].

Third, this review highlights the need for further methodological development in studies assessing the perception of physicians of the decision-making process within specific clinical encounters. None of the authors of the included instruments provided data on the responsiveness of their instruments (i.e. the extent to which the instrument measures physician change over time). Also, 'within physician' clustering of multiple data points (i.e. non-independence of data) produced statistical challenges that were dealt with inconsistently by their developers. In one instrument, clustering of multiple data point under each physician was taken into account for the factorial analysis but not for the reliability analyses [84,85]. Authors who took clustering into consideration used one of three strategies: average score per physician [55,56,59,74], one data entry per physician [47-53,75] or bootstrapping [57,64-67,69-71,84,85]. Therefore, methodological development in this area will be needed to ensure that responsive instruments and adequate analytical approaches are used in studies assessing the perception of physicians of the decision-making process within specific clinical encounters.

Lastly, for the included instruments, the mean number of items ranged from 6 to 37 items (mean = 16.7). It remains a challenge for health service researchers to develop sound measurements for conducting implementation studies that will minimize the burden to participating physicians. In our own experience, and in line with what has been reported in the literature, there appears to be an association between instrument length, defined in this systematic review as the number of items included in an instrument, and physician participation in studies [94]. This is perhaps even more apparent for health professionals' self-administered questionnaires after a specific clinical encounter. As such, our results provide some valuable insight or benchmarking about the number of items included in the instruments that are currently available for conducting studies on clinical decision-making with physicians.

This review has a number of limitations. Studies reporting the development of instruments are generally not well-indexed in electronic databases [95]. In this review, the search strategies used may not have been optimal even though we consulted with three experienced information specialists. It is possible that some eligible instruments as well as relevant publication regarding the included instruments were not included in this review. Also, clinical decision-making is moving from a unidisciplinary perspective to an interdisciplinary perspective [20]. Therefore, the included instruments might not be representative of on-going developments in healthcare decision-making. Indeed, recent health services policy documents clearly indicate the need for patient-centered care provided by an interprofessional team [96]. However, in a review on barriers and facilitators to implementing shared decision making in clinical practice as perceived by health professionals, the vast majority of participants (n = 2784) enrolled in the 28 included studies were physicians (89%) [97]. This suggests that more will need to be done to enhance an interprofessional perspective to shared decision making, a process by which a patient and his/her healthcare providers engage in a decision-making process. We firmly believe that the instruments that were identified throughout this review could be further developed using this interprofessional perspective.

Lastly, it is interesting to note that for the eleven included instruments, the mean score of items on the STARD was 12.4 (range: 2 to 18). It is important to emphasize that seven of the included instruments were published before the 2003 STARD criteria. Although, this mean score compared well to the mean scores of items on the STARD that were reported in test accuracy studies in reproductive medicine: 12.1, future research in this field will need to improve the reporting of the development of instruments that would assess healthcare professional's perspective of the decision-making process

## Conclusion

This systematic review provides valuable data on instruments that assess the perception of physicians of the decision-making process within specific clinical encounters. It can be used by educators and health services researchers as a repository of standardized measures of the physician perspective of the clinical decision-making process and we hope of other healthcare providers. It was not our intention to identify the "best" instrument but rather to offer options to the target audience. We believe that based on the context of its intended use, a process of weighting its limitations and strengths and other factors faced by its potential users, most if not all of the identified instruments might play a valuable role in the future. This systematic review also sent an important signal: in the XXI century, the clinical decision-making process might only be adequately assessed by using a dyadic approach. In this regard, some of the identified instruments might be more attractive than others. However, more research is needed to investigate the validation of these instruments. More specifically, for the production of evidence on the validity and reliability data of the instruments, analytical methods that take into account within physician clustering is required. For all the included instruments, the development of corresponding patient versions should be encouraged. The combined use of the patient version with its respective healthcare professional version will help capture the complexity of the clinical decision-making process and thus of its dynamic and reciprocal nature. Only then will a new and more comprehensive understanding of health-related decision-making in the context of specific clinical encounters be possible.

## Competing interests

All author(s) declare that they have no conflicting financial interests.

One of the authors (FL) is an author of three of the included studies.

## Authors' contributions

FL conceived the study, supervised KG and AL, validated the methods, validated the article selection, abstracted all included instruments, analysed the results, and wrote the paper. KG participated in the selection of the articles and abstracted all included instruments. KG and AL assessed the quality of the included studies and reviewed the paper. DM participated in the conception of the review, provided comments on the search strategy, validated the methods and reviewed the paper. GE validated the methods, participated in the interpretation of the results and reviewed the paper. All authors have read and approved the final version of the manuscript. FL is its guarantor.

## Pre-publication history

The pre-publication history for this paper can be accessed here:


